# Predicting accuracy in eyewitness showups: confidence and response time in the laboratory, confidence in the field

**DOI:** 10.1186/s41235-026-00734-w

**Published:** 2026-06-06

**Authors:** Laura Mickes, Karen L. Amendola, Xueqing Chen, Maria Valdovinos Olson, Curt A. Carlson, Scott Gronlund

**Affiliations:** 1 University of Bristol, Bristol, UK; 2 National Policing Institute, Arlington, VA USA; 3 East Texas A & M University, Commerce, TX, USA; 4 The University of Oklahoma, Norman, OK USA

**Keywords:** Eyewitness identification, Field identifications, Confidence–accuracy, Showups, Evidentiary strength, Confrontation, Response time, Reflector variables

## Abstract

Police administer live showup procedures when a suspect is located shortly after a crime, yet most prior research has relied on static, image-based showups, where confidence is often a weaker predictor of accuracy than in lineup procedures. Because actual police showup procedures involve live, in-person identifications, we conducted two studies to test the reliability of eyewitness confidence in live showups. In Study 1, a laboratory experiment (*N* = 229), identifications made with both high confidence and fast response time were more likely to be correct, and high-confidence rejections indicated suspect innocence. In Study 2 (*N* = 153), a field study using showup cases from two US jurisdictions, eyewitness confidence again indicated accuracy, assessed with the Strength of Evidence Scale as a proxy for ground truth. These findings demonstrate that eyewitness confidence is an indicator of accuracy in live showups, with implications for law enforcement policy and safeguards against wrongful convictions. When administered with procedures to reduce bias, live showups can yield reliable and probative identification, supporting their continued use in police investigations.

Eyewitness confidence in suspect identifications from lineups (also known as identification parades) was once thought to bear little relation to the accuracy of identification (e.g., Report of the Special Master, State of New Jersey v. Henderson, 2008). More recent evidence using newer methodology has reversed this view (e.g., Juslin et al., 1996; Mickes, 2015; Seale-Carlisle et al., 2024; Wells et al., 2020; Wixted & Wells, 2017). Findings now indicate that highly confident suspect identifications—*when expressed at the initial identification*—are typically accurate, whereas low-confidence identifications tend to provide inconclusive evidence or may reflect the misidentification of an innocent person. Importantly, this relationship has also been observed in applied contexts, including a field study conducted in Houston (W. Wells et al., 2016; Wixted et al., 2016).

The growing body of evidence on the relationship between confidence and accuracy has prompted several organizations to recommend policy changes. The International Association of Chiefs of Police (2016), the National Research Council of the National Academy of Sciences (2014), the US Department of Justice (Yates memo, 2017), and the American Psychology Law Society of the American Psychological Association^1^ (2020) have each issued guidelines or recommendations that law enforcement agencies record eyewitnesses’ initial^2^ confidence.

Police use identification procedures, such as lineups or showups (or field identifications or confrontations in the UK), to obtain evidence from eyewitnesses about a suspect. Lineups present the eyewitnesses with images of the police suspect (who may be innocent or guilty) alongside several fillers (individuals who resemble the suspect or match the description of the perpetrator but are known to be innocent). In contrast, showups present the eyewitnesses with the suspect alone. Understanding these procedural differences is needed for assessing how confidence reflects accuracy in each context.

Showups are typically conducted shortly after a crime, once the police have located an individual matching the perpetrator’s description. By contrast, lineups are typically administered after a longer delay. Another key difference is that showups are live, in-person procedures, whereas lineups are usually photographic arrays (though video lineups are used in the UK). During a showup, the eyewitness is brought to a location, usually near the crime scene, where the detained individual is held, to determine whether an identification can be made (Behrman & Davey, 2001). Showups are commonly conducted from the back seat of a police vehicle, preventing the potential suspect from seeing the eyewitness.^3^ The procedure is designed to allow the eyewitness to determine whether the detained individual is, might be, or is not the perpetrator.

Examining the confidence–accuracy relationship in field showups is important because they remain a common form of eyewitness identification practice. A national survey of eyewitness identification practices by the Police Executive Research Forum (PERF, 2013) found that field showups are conducted in over 60% of agencies, a proportion that is now just slightly higher (Amendola, Valdovinos Olson et al., 2025a and 2025b). This practice continues despite numerous recommendations from legal scholars, courts, and social and cognitive science researchers, as well as guidelines urging law enforcement agencies, courts, and legislators to restrict or largely avoid showups (e.g., Bertelsman, 2022; Brooks, 1983; Wells et al., 2020). Importantly, Amendola, Valdovinos Olson et al. (2025a and 2025b) reported that local police departments conduct showups at a higher rate (74%) than state agencies or sheriffs’ departments.^4^ The fact that over 60% of surveyed agencies continue to use showups may indicate either a lack of awareness of these recommendations or a reluctance to abandon a procedure seen as useful for investigations. Given their frequent use despite widespread concerns, it is necessary to examine how confidence functions in showups.

Although decades of research have advanced understanding of eyewitness identification, judicial decisions and field practices have often diverged from scientific evidence. Several factors contribute to this disconnect, including the rapid pace of scientific developments, inconsistent or unreplicated findings (often stemming from methodological or measurement issues), reliance on anecdotal evidence, counterintuitive results, and the well-documented time lag between scientific consensus and applied practice (e.g., Stoddard, 1935). Perhaps most importantly, a broader research-practice gap persists (e.g., Norman, 2010), driven in part by the mistaken assumption that scientists and practitioners share the same goals, understandings, or that they interact in meaningful ways. This broader gap highlights why research must assess not only accuracy in ideal laboratory conditions but also how findings translate to applied settings, such as with showups.

Scientific research on eyewitness identification has progressed rapidly, and in recent years its pace appears to have accelerated further. Expecting such rapidly advancing science to be quickly adopted in the field is unrealistic, given the well-documented slow diffusion of innovations (e.g., Green et al., 2009; Rogers, 1962). Although the exact length of the science-to-practice gap in psychology or criminal justice is unclear, research in the field of medicine suggests an average lag of approximately 17 years (e.g., Morris et al., 2011). One scholar in criminal justice has argued the lag in criminology may be at least that long, if not longer (Blomberg, 2021).

The broader research–practice gap significantly influences the extent to which courts and law enforcement professionals adopt scientific evidence (e.g., Suggs, 1979). A key factor is that tightly controlled laboratory research often cannot capture the complexities of field settings. As Norman (2010) noted, in practice, many variables “contradict the neat, tidy, logical assumptions of the scientist” (p. 11), reflecting longstanding concerns about the external and ecological validity of laboratory-based research.

Research on identification procedures has largely focused on one form of accuracy: discriminability, defined as the ability to distinguish innocent from guilty suspects. Studies consistently show that showups underperform relative to lineups, with participants demonstrating lower discriminability when memory is tested using showups compared to lineups (Key et al., 2015; Yarmey et al., 1994; collapsed across retention intervals, Yarmey et al., 1996; Gronlund et al., 2012; Mickes, 2015; Wetmore et al., 2015). Although much of the literature has emphasized discriminability, reliability provides a complementary and practically relevant measure of accuracy.

This other form of accuracy, which is the focus of the current paper, is reliability, operationally defined as the likelihood that an identified suspect is the true perpetrator at a particular level of confidence (Mickes, 2015). Reliability is assessed by plotting the proportion of correct identifications at each level of confidence in a confidence–accuracy characteristic (CAC) plot. When identifications are reliable, high-confidence suspect identifications are more accurate than medium-confidence suspect identifications, which in turn are more accurate than low-confidence suspect identifications. Understanding the reliability of identifications provides valuable information to triers of truth in making evidence-based decisions.

Much of the research on eyewitness identification has employed lineups. In laboratory studies, participants typically view a video of a perpetrator committing a mock crime and, after a distractor task, are tested on their memory of the perpetrator using a lineup. Participants may identify a suspect, a filler, or reject the lineup entirely (i.e., make no identification). Across most studies, confidence reliably predicts suspect identification accuracy (e.g., Wixted & Wells, 2017; Wixted et al., 2015), with high-confidence suspect identifications being more accurate than lower-confidence suspect identifications. Consequently, high-confidence identifications indicate a greater likelihood that the suspect is guilty, whereas low-confidence identifications are either inconclusive or suggest innocence. While this confidence–accuracy relationship is well documented in lineup studies, it is less clear whether the same pattern holds for showups.

In addition to the emphasis on lineups, research has largely focused on positive identifications (where the eyewitness selects a lineup member) rather than on lineup rejections (where no selection is made). Beyond examining how high-confidence positive suspect identifications can indicate guilt, recent research has explored whether high-confidence rejections signal innocence. Signal detection theory suggests that if high-confidence positive suspect identifications reflect guilt, then high-confidence lineup rejections should correspondingly indicate innocence (Yilmaz et al., 2024). The current research extends this question to showups, investigating whether showup rejections are similarly indicative of innocence.

Laboratory studies of showups follow procedures similar to lineup studies, but instead of selecting a perpetrator from a group of faces, participants view a single face. This face is either the individual depicted in the video (the guilty suspect, or “old”) or a different person (the innocent suspect, or “new”) and rate their confidence. The pattern of results for showups, using images, differs from that observed in lineups, where the confidence–accuracy relationship for showup identification decisions tends to be weaker. While high-confidence identifications tend to be more accurate than low-confidence ones, high-confidence identifications in showups have generally been found to be less accurate than high-confidence identifications in lineups (Carlson et al., 2021; Colloff & Wixted, 2020; Key et al., 2015; Mickes, 2015; Sauerland et al., 2018; Wilson et al., 2018). These findings call for theoretical accounts that can explain the discrepancy.

The diagnostic feature detection (DFD) account (Wixted & Mickes, 2014) explains the differences observed between lineups and showups. According to this account, confidence in showup decisions is less predictive of accuracy than confidence in lineup decisions because observers fail to recognize non-diagnostic features that should be discounted. When only a single individual is presented, rather than a lineup of individuals, it is not readily apparent that eyewitnesses should distinguish between diagnostic features that are relevant to prior exposure and non-diagnostic features that should be ignored.

Colloff and Wixted (2020) tested the DFD account  on a novel simultaneous showup procedure in which participants had access to diagnostic features. In this procedure, the suspect’s face was presented alongside other faces, but only the suspect’s face was designated as the one under consideration. The presence of additional faces allowed participants to distinguish between diagnostic and non-diagnostic features, resulting in improved performance. Specifically, high-confidence identifications in the simultaneous showup were more accurate than high-confidence identifications in a standard showup. Although including additional faces enhances memory performance in laboratory settings and advances theoretical understanding of eyewitness identification, it is impractical in the field, where showups are conducted as live, in-person procedures. Accordingly, it is important to turn to research that has examined showups in their real-world, live format.

Relatively little research has examined how the criminal justice system administers showups in the real world. Laboratory studies typically use photographic images as the stimuli, whereas police administer showups live in the field. A few live showup experiments have been conducted, though some did not allow for assessment of the confidence–accuracy relationship (e.g., Valentine et al., 2012). Eisen et al. (2017) compared live laboratory and simulated field showups by combining data from two live showup experiments and presenting a confidence–accuracy characteristic plot. High-confidence identifications from both laboratory-administered (by researchers) and field-administered (by police officers) studies ranged from 75 to 90% accuracy, values typically lower than those observed with lineups, but consistent with prior showup findings using photographic stimuli. In a later field experiment, participants made false identifications with high confidence in 50% of the cases (Eisen et al., 2022). In that study, suspects stood beside a police officer with their hands positioned as if handcuffed, making the procedure highly suggestive. By contrast, in laboratory-based live showups, 27% of false identifications were made with high confidence. Together, these findings suggest that confidence is less predictive of accuracy when memory is tested through live showups and may not differ substantially from image-based showups.

In addition to confidence, response time has been shown to be an informative indicator of accuracy in eyewitness identification. In lineup studies, faster suspect identifications tend to be more accurate than slower ones (e.g., Seale-Carlisle et al., 2019), making response time, like confidence, a useful marker of reliability. Similar patterns have been observed in showup research. Eisen et al. (2022) measured response times during live showups and found that accurate identifications were faster than inaccurate ones. Using photographic showups, Sauerland et al. (2018) reported that high-confidence identifications made quickly were especially accurate, more so than both lower-confidence identifications and high-confidence identifications made slowly. Recently, Carlson et al. (2025) found correct identifications to be faster than false identifications for biased and fair photographic lineups and image-based showups. The most accurate identifications from both lineups and showups were fast and made with high confidence.

The lack of experiments on live showups is likely due to practical constraints. Collecting data from hundreds of participants is resource-intensive, time-consuming, and costly. In laboratory settings, researchers can easily use face databases to obtain innocent suspect and filler images that resemble the perpetrator, whereas creating equivalent live procedures poses greater challenges. For police, however, showups are comparatively efficient: They can be conducted within minutes or hours after the crime, require no fillers, and are less time-consuming than lineups (Gronlund et al., 2012; Valentine et al., 2012). Despite their inherent suggestiveness, given the suspect is always obvious, showups remain widely used, with over 60% of agencies reporting reliance on them (PERF, 2013; Amendola, Valdovinos Olson et al., 2025a and 2025b). This combination of practical appeal and widespread use underscores the need for research on live showups, both to advance theoretical understanding and to inform practice.

## Study 1: laboratory study

The present investigation addressed two primary questions:

1. Does confidence in a showup identification, or in a rejection, increase the probative value of eyewitness evidence?
2. Does response time in a showup identification similarly increase the probative value of eyewitness evidence?

Drawing on findings from a recent live laboratory showup experiment (Seale-Carlisle et al., 2026), we predicted that both confidence and response time would provide meaningful predictive information about identification accuracy.

## Method

### Sample size and participants

#### Sample size considerations

Because our analyses rely on graphical methods (CAC and RAC curves), sample size requirements are driven by the need for stable estimates within confidence and response–time bins rather than by formal power analyses. We based our target sample size on prior CAC research, particularly the Houston Police Department Field Study (W. Wells et al., 2016; Wixted et al., 2016), which demonstrated stable confidence–accuracy patterns using a sample size in the range of ~ 200 participants, providing a benchmark for the present design.

#### Participants

A total of 229 participants were recruited at the University of Bristol Neuroscience Festival in March 2023 and were randomly assigned to either a target-present showup (*n* = 120) or a target-absent showup (*n* = 109). Ages ranged from 8 to 73 years (*M* = 28, *SD* = 14.57). Participants under 16 years of age (*N* = 18, *M* = 12; *SD* = 1.97) were accompanied by their legal guardians, who provided signed consent prior to participation. Ethical approval was obtained from the University of Bristol Research Ethics Committee (reference number 13830). Table 1 shows the participant characteristics.

**Table 1:** Participant characteristics (*N* = 229)

	*N*	*%*
Gender		
Female	154	67
Male	72	31
Unknown	3	1
Race/Ethnicity^a^		
White	180	79
Asian	27	12
Black	4	2
Hispanic	4	2
Other	13	6

Totals may not add up to 100 because of rounding

^a^Three participants did not provide their ethnicity

### Materials

#### Suspects

Ten actors served as the suspects. Four Asian students (2 female and 2 male) volunteered as research assistants and were assigned as actors, while six White students (3 female and 3 male) responded to an advertisement and passed the screening. All actors were young adults with similar physical features. Male actors were required to have: (1) short brown or dark hair; (2) height ranging from about 5 ft, 6½ in. to 6 ft (172–183 cm); (3) weight ranging from about 165–185 lbs. (75–84 kg); (4) light or brown eye color; (5) shaved face with no beards, mustaches, etc.; and (6) no distinctive features (e.g., no tattoos, piercings, or scars). Female actors were required to have: (1) medium to long, brown or dark hair; (2) height ranging from about 5 ft, 2½ in. to 5 ft 5 in. (160–168 cm); (3) weight ranging from about 110–141 lbs. (50–64 kg); (4) light or brown eye color; and (5) no distinctive features.

To ensure participants focused on memory for the person rather than clothing, all actors wore the same black Bristol Neuroscience Festival T-shirt and a black hat during the study phase, and their own plain hoodies without a hat during the test phase. Male actors wore blue jeans, and female actors wore black leggings throughout the experiment. All actors wore white sneakers and shoe covers.

#### Venue

**Fig. 1:**
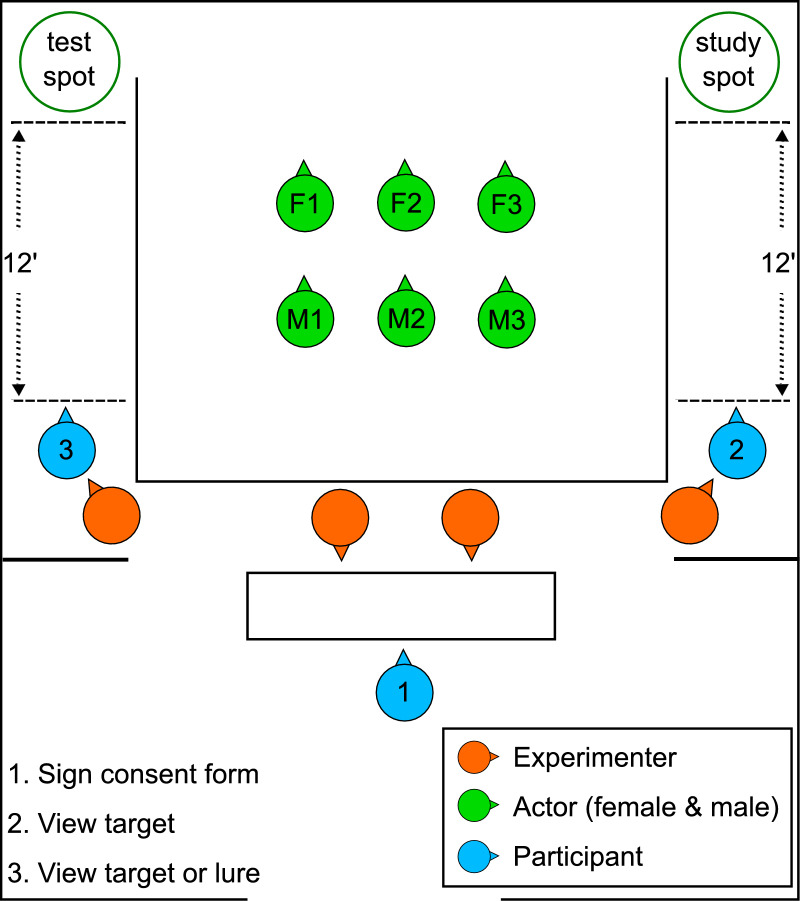
Top-down view of the experimental setup for study 1. *Note.* Participants first signed a consent form (1), were taken to the study spot for the study phase (2), and walked to the test spot for the test phase (3). F1, F2, and F3 indicate some of the female actors and M1, M2, and M3 indicate some of the male actors

As shown in Fig. 1, a makeshift room made of movable partitions was used to house the actors, preventing visibility to other festival attendees. The marked spots are where the actors stood. Participants observed the actors from 12 feet at both the study (for 10 s) and test spots (until the participant responded). In target-present showups, the same actor appeared at both the study and test locations (i.e., the guilty suspect). For the target-absent showups, actors of the same gender and race were randomly paired, with one actor appearing during the study phase and the other during the test phase (i.e., the innocent suspect). A counterbalanced actor list ensured an equal number of target-present and target-absent showups, and all actors were assigned as targets an equal number of times.

### Procedure

Eyewitness identification studies typically have three phases: study, distractor, and test. The study phase simulates the eyewitness encoding the crime. The distractor phase represents the interval between the commission of the crime and the identification procedure. Finally, the test phase corresponds to the identification procedure during which the eyewitness attempts to identify the perpetrator.

#### Study phase

Following consent, participants were guided to the study-marked spot (blue icon labeled 2 in Fig. 1). Adult participants were instructed, “*You are about to see a person for many seconds. Please try to remember what they look like*.” Minor participants received slightly adapted age-appropriate instructions (to increase engagement), “*A ‘baddie’ will appear at that spot for a few seconds. Please try your best to remember their face, but not their clothes or shoes*.” Participants confirmed their understanding before an actor, portraying the perpetrator, stood at the study spot silently counting to ten, facing forward with a neutral expression.

#### Distractor phase

Participants answered demographic questions and engaged in a 5-min activity. Adults discussed memory-related brain regions and their festival experiences with research assistants. Minor participants also played a backward-alphabet game.

#### Test phase

Participants were then escorted to the test-marked spot (blue icon labeled 3 in Fig. 1) and received age-appropriate instructions. Adult participants were informed, “*You are about to see a person. The person you saw earlier may or may not be the person you are about to see. If the person is the same as earlier, please circle ‘yes’ on your answer paper. Otherwise, circle ‘no’*.” Minor participants were told, “*You will see a person at that spot. They may or may not be the baddie you saw before. If they are, circle ‘yes’ on your answer paper; otherwise, circle ‘no’*.”

After confirming that participants understood the instructions, either the same actor (target/guilty suspect) or a different actor of the same race and gender (lure/innocent suspect) stood at the test spot, facing forward with a neutral expression to allow for clear observation. Participants first indicated whether the suspect at the test spot was the same person seen at the study spot (“old”) or a new person. They then rated their level of confidence. A research assistant recorded response time, starting when the suspect appeared and stopping when the participant made a decision. Participants then rated their confidence in the decision using a 6-point scale: *definitely new, probably new, guess new, guess old, probably old, definitely old*. The confidence scale was interpreted as follows:

- *Definitely new*: Participant believes the suspect is not the perpetrator.
- *Probably new*: Participant believes the suspect may not be the perpetrator.
- *Guess new*: Participant is guessing the suspect is not the perpetrator.
- *Guess old*: Participant is guessing the suspect is the perpetrator.
- *Probably old*: Participant believes the suspect may be the perpetrator.
- *Definitely old*: Participant believes that the suspect is the perpetrator.

Finally, all participants were debriefed.

### Analysis

#### Analysis plan

Study 1 analyses were conducted using pyWitness (https://lmickes.github.io/pyWitness/; Mickes et al., 2023), and the data and analysis code are available at https://osf.io/9rhkv/overview?view_only=215e9c25f6674a7cb4056fb7a9e0f9c3. The analyses of interest were confidence–accuracy characteristic (CAC) and response–time accuracy characteristic (RAC) curves (Mickes, 2015; Seale-Carlisle et al., 2019). Both CAC and RAC analyses provide measures of reliability, estimating the likelihood that a suspect identified in the showup is the true perpetrator. CAC curves reflect accuracy as a function of confidence level, while RAC curves reflect accuracy as a function of response time. If confidence is not predictive of accuracy, the CAC curve is expected to be flat; if it is predictive, a positive slope is expected for choosers (participants who identified the suspect) and a negative slope for non-choosers (participants who rejected the showup). Likewise, if response time is not predictive of accuracy, the RAC curve should be flat, whereas a negative slope indicates predictive value. To examine these relationships, confidence and response time were grouped into categories, and the proportion of correct identifications within each category, or bin, was plotted to generate the CAC and RAC curves, respectively.

To test our first hypothesis, that confidence predicts accuracy, confidence ratings to positive identifications were grouped into four categories for analysis: *definitely new, probably new, probably old, definitely old*. Only two “guess” responses were made (one guess new, one guess old), which were collapsed into the adjacent categories (*guess new* with *probably new, guess old* with *probably old)*. Accuracy for positive IDs was then calculated for each confidence bin using the formula:

$${\mathrm{CAC}} = \frac{{{\mathrm{CID}}_{{{\mathrm{conf}}}} }}{{{\mathrm{CID}}_{{{\mathrm{conf}}}} + {\mathrm{FID}}_{{{\mathrm{conf}}}} }}$$

where CID_conf_ is the number of correct suspect identifications at a given level of confidence from target-present showups, and FID_conf_ *is* the number of false identifications made with a level of confidence from target-absent showups. CAC values are then plotted to generate the CAC curves.

To assess whether high-confidence rejections are more diagnostic of suspect innocence than low-confidence rejections, we also computed showup rejections by adapting the CAC formula for showup rejections. Specifically, correct rejections from target-absent showups were treated as accurate responses, whereas misses from target-present showups were treated as inaccurate. Accuracy for each confidence bin was then calculated using the CAC formula using the confidence–accuracy characteristic formula by adapting it for rejections. Thus, accuracy for showup rejections is computed for each confidence bin using the formula:

$${\mathrm{CAC}} = \frac{{{\mathrm{CR}}_{{{\mathrm{conf}}}} }}{{{\mathrm{CR}}_{{{\mathrm{conf}}}} + {\mathrm{MR}}_{{{\mathrm{conf}}}} }}$$

where CR_conf_ is the number of correct rejections at a given level of confidence from target-absent showups, and MR_conf_ is the number of misses made at a level of confidence from target-present showups. CAC values are then plotted to generate the rejection-based CAC curves.

To test our second hypothesis, that response time predicts accuracy, response times were categorized from slow to fast for analysis. RAC values were then calculated for each bin using the formula:

$${\mathrm{RAC}} = \frac{{{\mathrm{CID}}_{rt} }}{{{\mathrm{CID}}_{rt} + {\mathrm{FID}}_{rt} }}$$

where *rt* is response time, *CID*_*rt*_ is the number of correct identifications at a given response–time bin from target-present showups and *FID*_*rt*_ is the number of false identifications at that response–time bin from target-absent showups. The bins were constructed to contain approximately equal numbers of responses, resulting in the following intervals: 0–2, 2–3, 3–4, 4–10, and > 10 s. RAC values were then plotted to generate the RAC curves.

### Results

**Fig. 2:**
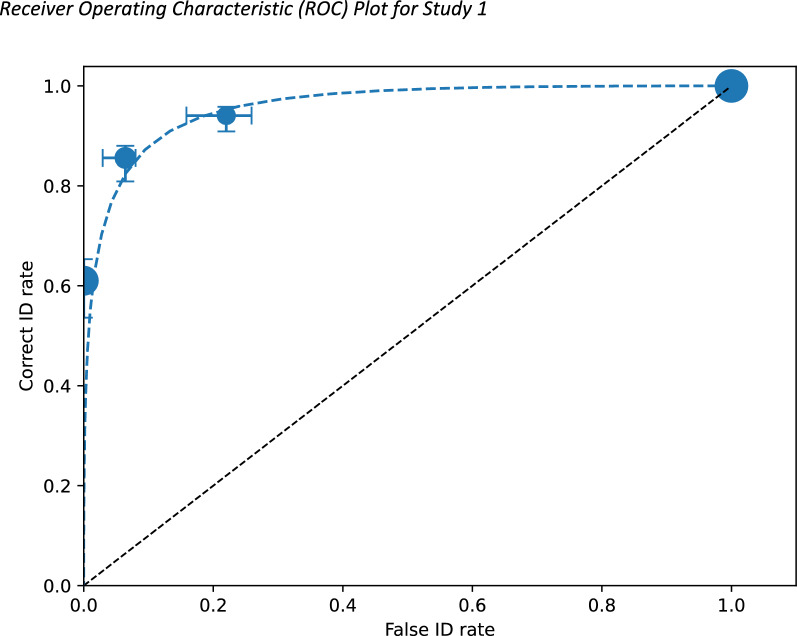
Receiver Operating Characteristic (ROC) plot for study 1. *Note*. Error bars represent 68% confidence intervals from 200 bootstrap samples. Point sizes indicate relative frequency. The black dashed line represents chance performance. The blue dashed curve shows the fit of the equal variance Independent Observations signal detection model, which was excellent (χ^2^ = 2.476, *p* = 0.290)

To examine the reliability of eyewitness identifications in the showup procedure, we first assessed overall discriminability (using correct and false identification rates) and then analyzed the data using CAC and RAC curves. The overall correct identification rate was 0.856 and the false identification rate was 0.064. Table 2 shows the number of identifications by response type for target-absent and target-present showups at each confidence level. Discriminability was high, as shown by the ROC curve in Fig. 2, with area under the ROC curve = 0.95 (*d′* = 2.58). These values indicate that participants had a strong ability to distinguish the guilty suspect from an innocent person, as reflected by the high discriminability shown in Fig. 2.

**Table 2:** Number of responses by response type for target-absent and target-present showups by confidence level for study 1

	Confidence					
	Definitely new	Probably new	Guess new	Guess old	Probably old	Definitely old
Target-absent	85	17	0	0	7	0
Target-present	7	9	1	1	28	72

**Fig. 3:**
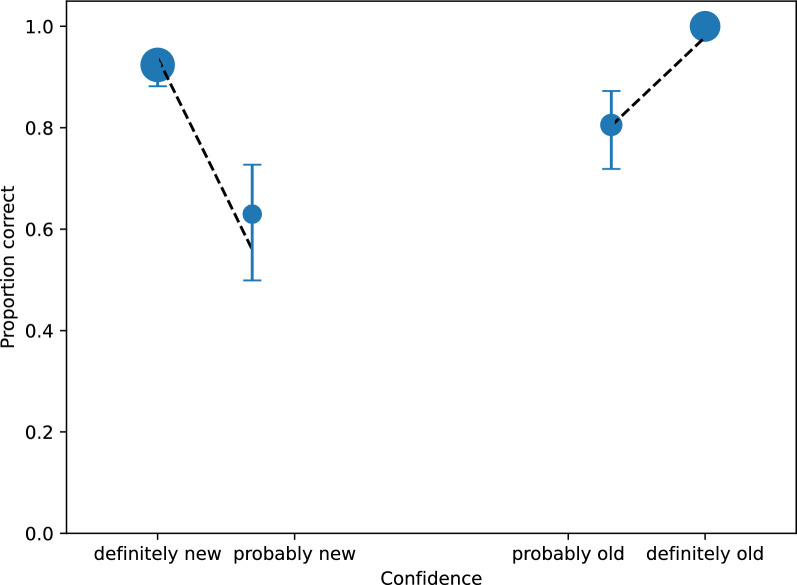
Confidence–Accuracy Characteristic (CAC) curve for positive identifications (“Old”) and showup rejections (“New”) for Study 1. *Note.* The error bars are 68% confidence intervals based on 200 bootstraps. The point sizes represent relative frequency. Due to low frequencies, guess responses were collapsed into adjacent categories

Figure 3 shows the CAC curve. For the positive identifications and rejections (responses in which participants correctly indicated that the suspect was not the perpetrator in target-absent showups), the highest confidence responses are more accurate than the lower-confidence responses. The *definitely old* responses were higher than the *definitely new* responses, which is consistent with the basic memory literature (e.g., Mickes et al., 2011; Tekin et al., 2021).

**Fig. 4:**
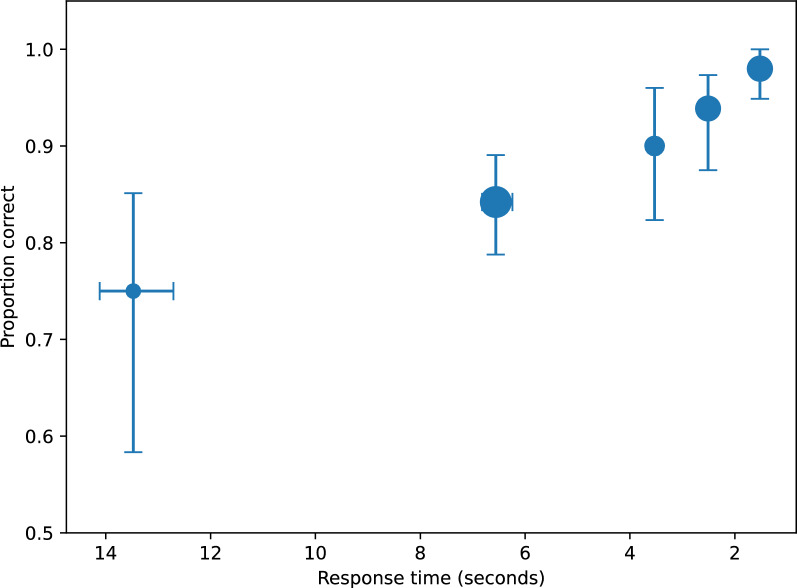
Response Time Accuracy Characteristic (RAC) curve for study 1. *Note.* The error bars are 68% confidence intervals based on 200 bootstraps. The point sizes represent relative frequency

Figure 4 shows the RAC curve. Accuracy tended to be higher for faster responses compared with slower responses. Overall, the pattern is approximately linear, suggesting that quicker responses were generally associated with greater accuracy. Together, these results suggest that higher-confidence identifications are generally associated with greater accuracy, as are high-confidence rejections in showup procedures. Likewise, faster response times are generally associated with greater accuracy.

### Discussion

In this study, we tested the hypotheses that confidence and response time would indicate accuracy in live showups. Our findings confirmed both predictions: Identifications made with higher confidence were more accurate than those made with lower confidence, and identifications made more quickly were more accurate than those made more slowly. High-confidence identifications were highly accurate (100%). Interestingly, the same pattern applies to showup rejections, with high-confidence rejections being highly accurate (92%), suggesting a strong signal of suspect innocence. Together, these results extend the well-documented ability for confidence and response time to indicate accuracy from lineup research into the underexplored domain of live showups.

Participants demonstrated strong discriminability between guilty and innocent suspects, as reflected in the ROC analysis (Fig. 2). One consideration, however, is that overall performance in Study 1 was relatively high, with accuracy at the highest confidence levels approaching ceiling (Fig. 3). Under more challenging viewing conditions (such as shorter exposure durations), overall memory performance would likely be lower. However, prior research indicates that the confidence–accuracy relationship, particularly for high-confidence identifications, remains robust even when overall discriminability is reduced (e.g., Wixted & Wells, 2017). Thus, although absolute accuracy levels may vary across conditions, the pattern observed here, where higher confidence corresponds to higher accuracy, is expected to generalize beyond the present experimental context.

To understand why our findings differ from image-based showups, we turn to theoretical explanations. Live and image-based showups reveal different patterns in the relationship between confidence and accuracy. In most showup experiments using images, confidence tends to be less predictive of accuracy than in lineup studies. Diagnostic Feature Detection (DFD) theory (Wixted & Mickes, 2014) provides one account of this pattern, proposing that when eyewitnesses view only a single face, they are less able to distinguish diagnostic from non-diagnostic features, resulting in lower discriminability and flatter CAC profiles.

In contrast, the CAC patterns observed in the present study more closely resembled those found in fair, image-based lineup procedures. One possible explanation is that live showups provide richer and more ecologically valid perceptual information than static images, allowing witnesses to form more detailed and diagnostic representations of the face. Factors such as depth, motion, and naturalistic viewing conditions may enhance encoding and retrieval, thereby improving discriminability. These features of live showups may help explain why confidence was more strongly related to accuracy in the present study than in prior image-based showup research.

Importantly, our results also showed that rejections were accurate when participants were highly confident that the suspect was not the perpetrator. This finding highlights that confidence provides useful information not only for positive suspect identifications but also for rejecting innocent suspects, which is particularly important for safeguarding against wrongful accusations.

It is also possible that participants in our experiment benefited more from the encoding specificity principle (Tulving & Thompson, 1973) than they would from image-based showups. The encoding specificity principle holds that memory is improved when the stimuli presented during encoding are similar to those presented during retrieval phases. The encoding specificity principle may have contributed to the strong performance observed in our live showups. In our experiment, with live showups, the stimuli (the actors) were shown standing at the same distance from participants during encoding and retrieval (though they moved to a different location for the test phase; see Fig. 1). In typical image-based showup studies, participants view a mock crime and are later shown an image of a face that may depict the innocent or guilty suspect. The overlap between study and test conditions in our study may have boosted recognition in a way that image-based showups cannot match.

Another potential explanation is that live showups provide richer perceptual information than images. Wetmore et al. (2015) suggested that in-person procedures may offer additional retrieval cues, potentially enhancing eyewitness performance relative to image-based presentations. To minimize reliance on non-facial cues, actors in the present study changed clothing between the study and test phases and were positioned in a different location at test (see Fig. 1). Nevertheless, the live context may have provided participants with subtle perceptual or contextual cues that supported recognition.

It is important to interpret this possibility cautiously. Research comparing live and photographic lineups has generally found little consistent evidence for a robust live-superiority effect, suggesting that increased realism does not necessarily translate into improved identification accuracy (e.g., Fitzgerald et al., 2018). Thus, the present findings should not be taken as evidence that live procedures are inherently superior to image-based ones. Rather, the stronger confidence–accuracy relationship observed here may reflect a combination of factors, including the use of live stimuli, differences in overall discriminability, and other features of the experimental design. Future research directly comparing live and image-based showups within the same paradigm will be important for isolating the mechanisms underlying these differences.

As in lineup studies (e.g., Brewer et al., 2006; Dunning & Perretta, 2002; Holdstock et al., 2022; Seale-Carlisle et al., 2019), quicker identifications were generally more accurate than slower ones. Our findings align with previous showup experiments, including image-based showups (Sauerland et al., 2018) and live showups (Eisen et al., 2022). If replicated consistently, this pattern suggests that law enforcement could benefit from recording response times during showups. However, it is worth noting that in real-world field settings, recording precise response times can be challenging. For example, timing may be affected by the eyewitness attempting to speak while being transported to the suspect’s location—a complication reported by at least two agencies.

### Limitations

To keep participants engaged, we limited the distraction to five minutes. This design choice, however, may reduce ecological validity, as real eyewitnesses often experience longer retention intervals before making an identification. Future studies should examine reliability (using CAC and RAC) in live showups with longer delays to better approximate police procedures.

A second limitation is that participants were aware they were taking part in an experiment, and their decisions carried no real consequence for the suspect. Unlike real eyewitnesses, participants likely experienced little arousal or stress, factors that may influence encoding, storage, and retrieval. To address this gap, there is a need for research conducted in partnership with law enforcement that involves actual eyewitnesses to real crimes. We attempted to bridge this gap by conducting a controlled laboratory study and a field study.

### Conclusions

We investigated the reliability of live showup identifications in a controlled laboratory experiment. The findings indicate that identifications from live showups can be informative when confidence and response time are considered. This study adds to a small but growing body of research on live showups and carries important applied implications. Although showups are often regarded as highly suggestive, they remain a common tool for law enforcement, as noted in the PERF Report (2013) and in the National Policing Institute’s nationwide survey (Amendola, Valdovinos Olson et al., 2025a and 2025b). Evidence that live showups yield meaningful indicators of accuracy should provide some reassurance regarding their continued use. If similar results are observed across a wider set of studies, identifications from showup procedures may be more reliable than is often assumed, even amid ongoing concerns about their suggestibility^5^. While our laboratory findings provide evidence that confidence and accuracy are useful indicators of accuracy in live, controlled showups, an important next step is to examine whether these relationships hold in actual police practices.

Ultimately, the reliability of eyewitness memory in showup procedures depends on key indicators, such as confidence and response time. Recognizing these factors allows researchers and practitioners to move beyond the question of whether showups are reliable, toward understanding when and under what conditions they can be used reliably. In the next section, we extend this inquiry to a police archival study to examine how confidence and response time indicate accuracy in real-world cases.

## Study 2: field study

In Study 2, we examined whether confidence and response time can serve as indicators of accuracy in real-world police showups. Laboratory studies allow for experimental control over variables, but they cannot fully capture the stakes, stress, and complexity of real criminal investigations. Archival data from two US jurisdictions, Denver, Colorado, and Hartford, Connecticut, were analyzed to determine whether reliability observed in the laboratory extends to applied settings.

Field research poses two main challenges. First, few agencies systematically record eyewitness confidence during showups, making such data difficult to obtain. Second, establishing ground truth in real cases is rarely possible, even when DNA evidence is available. To address these challenges, we recruited two agencies that we knew had been collecting confidence for over two years, and we used the Strength of Evidence Scale (SES; Amendola & Slipka, 2009) as a proxy for suspect guilt, independent of eyewitness identification. Additionally, while Study 1 demonstrated that response time indicates accuracy in live showups, we could not assess this factor in the field because agencies did not consistently record when eyewitnesses began making their identifications.

Based on the findings from Study 1 (and Seale-Carlisle et al., 2026), we hypothesized that confidence would provide meaningful information about the accuracy of eyewitness identifications in real-world showup procedures. Seale-Carlisle et al. (2026) similarly found that, in a live showup study, higher confidence was associated with greater accuracy, suggesting that these cues may generalize beyond traditional lineup procedures.

### Method

#### Agency sites

**Fig. 5:**
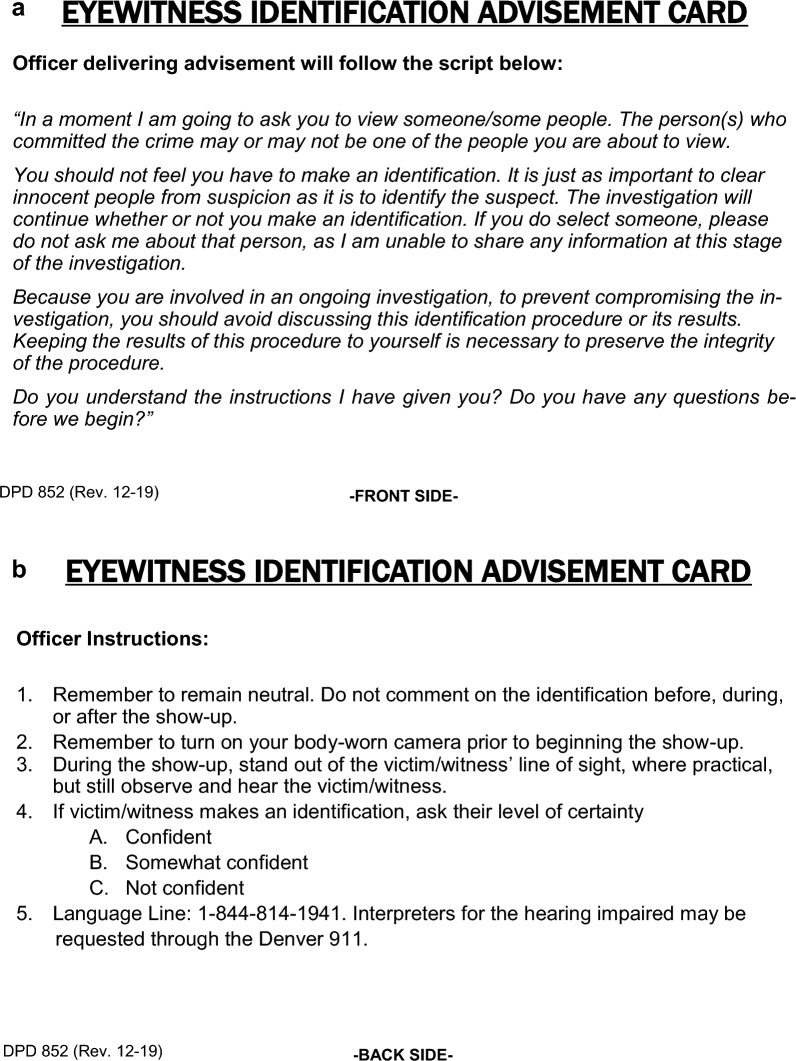
**a** Denver lineup or showup script for advising witnesses. **b** Denver instructions to officer(s) administering the lineup or showup. *Note.* Although item 4 instructs officers to ask eyewitnesses for their level of certainty, in practice, eyewitnesses were asked to provide their level of confidence in their own words. These statements were subsequently coded into confidence levels

**Fig. 6:**
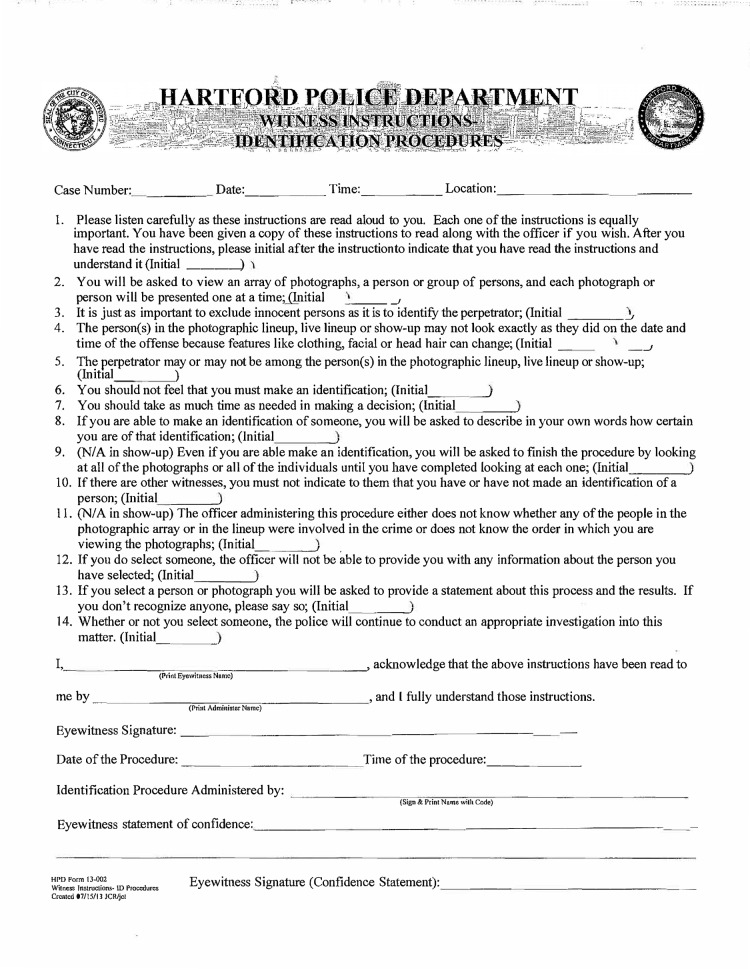
Hartford instructions to witnesses

Two jurisdictions that systematically collect eyewitness confidence in showups, the City and County of Denver, Colorado (*n* = 73), and Hartford, Connecticut (*n* = 80), served as research sites. These sites were selected because their police departments and state’s attorney office (Hartford) had sufficient case volume, accessible records, and standardized protocols for capturing confidence statements that minimized bias. In Denver, officers use an advisement card and read instructions to eyewitnesses (as shown in Fig. 5a) and the officers are also provided with instructions to follow (as shown in Fig. 5b). In Hartford, officers use standardized instructions across showups and lineups (as shown in Fig. 6).

### Data access

Archival police records were used to preserve real-world conditions. Data were obtained directly from both departments, and in Hartford, supplemented with an on-site review at the State’s Attorney’s Office.

#### Case availability

Initial case counts suggested sufficient showup data, but many files were unusable due to missing or incomplete confidence statements, state restrictions (e.g., juvenile cases), expungement, or inadequate documentation. While such limitations reduced the usable sample, they reflect the realities of conducting applied research with police records.

#### Response times

As is typical in most law enforcement agencies, neither jurisdiction consistently recorded eyewitness response times, limiting analysis of this variable in the field. Officers noted practical challenges, such as witnesses making spontaneous statements before formal questioning, which complicated the capture of response times in practice.^6^

### Overcoming the “ground truth” problem

**Fig. 7:**
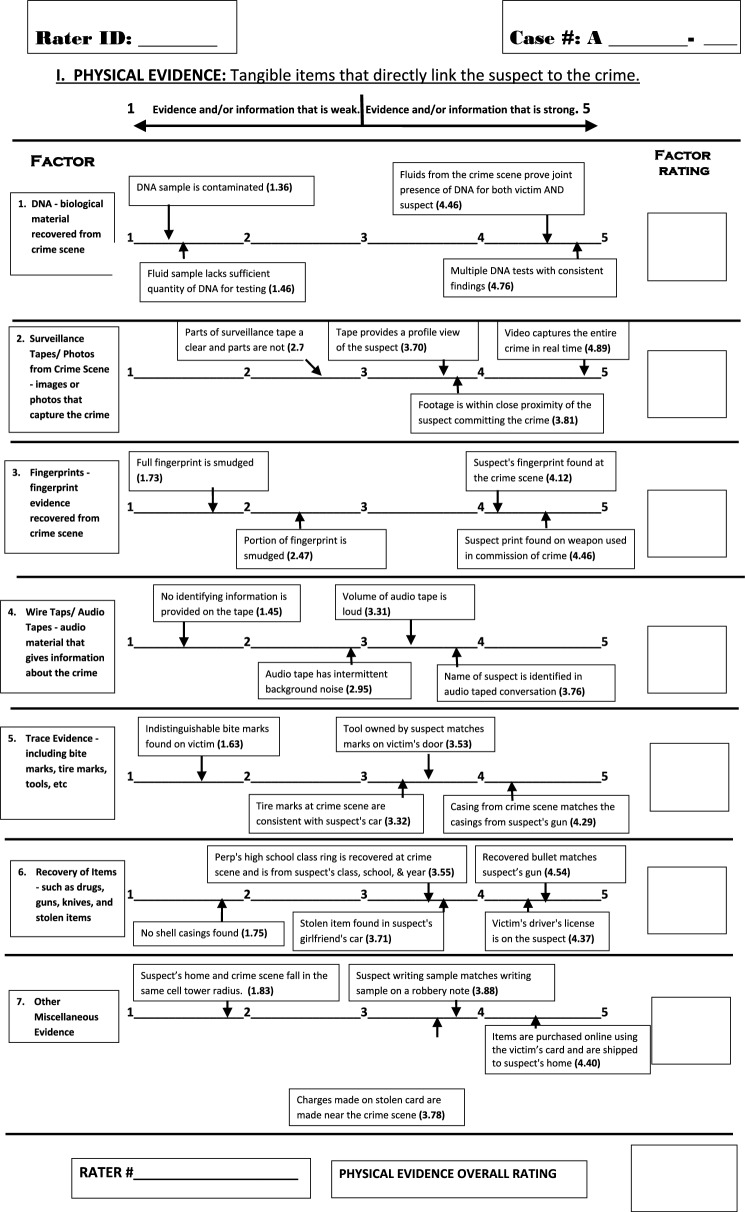
Physical evidence rating scale, from strength of evidence scale (SES; Amendola & Slipka, 2009)

To estimate ground truth in the field, we used the SES (Amendola & Slipka, 2009). The SES is a rating system that allows experts to evaluate the strength and quality of evidence in a criminal case across six categories: physical evidence, suspect statement, suspect history, victim characteristics, witness characteristics, and identification information. Each category is rated on a 5-point scale, ranging from 1 (weak evidence) to 5 (strong evidence), with the midpoint representing evidence of moderate strength. Using this instrument reduces subjective bias and provides a proxy for actual guilt or innocence, informed by the expertise of police, prosecutors, defense attorneys, and judges. Figure 7 shows an example of the rating scale for physical evidence.

### Study protocols

For this field study, SES ratings provided corroborating evidence, regardless of admissibility in court, to increase the diagnosticity of the analysis. To reduce selection bias, we included both successful showups (where the suspect was identified) and unsuccessful showups (where the suspect was not identified). This allowed us to examine a range of eyewitness decisions, from no identification to identifications made with low, moderate, or high confidence.

#### Obtaining and coding confidence statements

Following the Houston Police Department field study (W. Wells et al., 2016), we used a simple confidence scale to obtain confidence ratings. Members of the research team served as coders of the confidence statements. Where discrepancies of 1 point or more were present, two senior members of the research team re-evaluated the cases, and a final senior researcher resolved the consensus process by averaging the final ratings. In both sites, eyewitness confidence statements were collected in their own words and then coded into a 4-point scale:

1 = definitely not or not the person

2 = possibly the person/somewhat confident

3 = probably the person/confident to very confident

4 = definitely the person/highly confident (100% sure)

#### Protocols for rating evidentiary strength

Case evaluators rated the strength of the corroborating evidence using the SES as a proxy for ground truth, following procedures similar to the Austin Field Study (Amendola & Wixted, 2015), and whose reliabilities are reported elsewhere (Amendola & Wixted, 2015; Gould et al., 2012; Katzman & Kovera, 2022). Two former (recently retired) prosecutors from one US jurisdiction^7^ independently evaluated each case using the five-point scale.

Evaluators completed a four-hour training facilitated by two members of the research team that covered:

- SES procedures
- Evidence categories (dimensions)
- Types of evidence within each category (factors)
- Rating scale anchors and associated point values (i.e., exemplars)
- Efficient evaluation methods

Training concluded with practice cases, after which evaluators discussed their ratings to calibrate approaches. These same two prosecutors evaluated cases across both agencies to ensure consistency and interrater agreement. By training these two raters together in a training and practice session, we anticipated closer calibration of ratings, based on shared understandings of the rating scale and its usage.

There are three methods for establishing interrater agreement (Stemler, 2004), and we relied on the consensus method. This consensus-based approach prioritizes rating accuracy and shared understanding over independent agreement metrics. Because final ratings reflect consensus judgments, traditional interrater reliability coefficients were not computed.

Evaluators’ initial ratings were sent to two research team members who facilitated a structured consensus discussion to resolve discrepancies of two points or more (e.g., when one rated evidence as weak and the other as strong). Post-discussion, each evaluator provided their final rating, which was recorded by research team members, who then averaged the final scores. In both jurisdictions, case evaluators received redacted case files, including police reports, dispositions, and information indicating that a showup was conducted, without any personal information that could bias their assessment of the strength of the corroborating evidence.^8^

### Analysis

#### Sample size considerations

As in Study 1, sample size requirements were guided primarily by the need for stable estimates within confidence bins for CAC analyses rather than formal power calculations. Our initial target of approximately 350 showups was a planning estimate based on the anticipated number of usable cases across three participating jurisdictions (approximately 125 cases per site). One site was later unavailable, leaving two final research sites.

Following data collection, we conducted a post hoc sensitivity analysis for a correlation to provide a benchmark for the effects detectable with our sample size. With a minimum of *N* = 125, a two-tailed test with *α* = 0.05 and power of 0.80 provides sensitivity to detect effects of approximately *r* ≈ 0.22 (Cohen, 1988). This indicates that the study was adequately powered to detect small-to-moderate associations between confidence and evidentiary strength.

After exclusions, we retained 80 showups from Hartford (2014–2021) and 73 from Denver (2021–2023), yielding a total of 153 usable cases. Although smaller than the initial planning estimate, this sample was sufficient to produce stable CAC patterns and to examine the relationship between confidence and evidentiary strength. Accordingly, we proceeded with the planned analyses while interpreting the results with caution.

#### Analysis plan

CAC analysis was conducted to evaluate how confidence corresponded to case outcomes, using evidentiary strength ratings from the SES (Amendola & Slipka, 2009) as a proxy for ground truth. High guilt ratings (4–5) and low guilt ratings (1–2) were used to compute the likelihood of guilt across confidence levels, visualized via CAC curves.

To complement the CAC analysis, we computed the Adjusted Normalized Resolution Index (ANRI; Yaniv et al., 1991), which provides a single-value summary of how well confidence discriminates between correct (suspect likely guilty) and incorrect (suspect likely innocent) identifications. ANRI values range from 0 to 1, with higher values indicating greater discrimination. This measure was included as a secondary summary of the confidence–accuracy relationship, particularly given the smaller and more variable nature of field data. Finally, correlations were used to quantify the relationship between eyewitness confidence and evidentiary strength, allowing us to estimate the extent to which higher confidence was associated with stronger corroborating evidence of guilt.

To maintain consistency with Study 1, we present the CAC analyses first in the Results section, followed by the ANRI and then the correlation analysis. This combination of descriptive, graphical, and inferential approaches provides converging evidence regarding the reliability of confidence as an indicator of suspect guilt in real-world showups.

### Results

The data are available at https://osf.io/9rhkv/overview?view_only=215e9c25f6674a7cb4056fb7a9e0f9c3. Table 3 shows the number of identifications by response type for target-absent and target-present showups at each confidence level. Because ground truth is not directly observable in field data, evidentiary strength ratings from the SES were used as a proxy: Cases rated low in evidentiary strength (1–2) were treated as more likely to involve innocent suspects, whereas cases rated high in evidentiary strength (4–5) were treated as more likely to involve guilty suspects.

**Table 3:** Number of responses by confidence level for study 2

	Confidence			
	Rejection	Low	Moderate	Very high
Presumed to be:	Definitely not the person (1)	Possibly the person (2)	Probably the person (3)	Definitely the person (4)
Target-absent (low SE)	13	6	1	7
Target-present (high SE)	4	4	35	72

SE = Strength of evidence

We created CAC curves to examine the relationship between confidence and the likelihood of guilt. The likelihood of guilt was calculated as

$${\mathrm{Likelihood}} {\mathrm{of}} {\mathrm{Guilt}} = \frac{{\text{High Guilt Ratings}}}{{{\text{High Guilt Ratings}} + {\text{Low Guilt Ratings}}}}$$

where “High Guilt” was defined as evidentiary strength ratings of 4–5 (strong evidence) and “Low Guilt” as ratings of 1–2 (weak evidence). Cases rated as 3 or 3.5 were excluded due to their inherent ambiguity, representing 11 out of the 153 total cases.

**Fig. 8:**
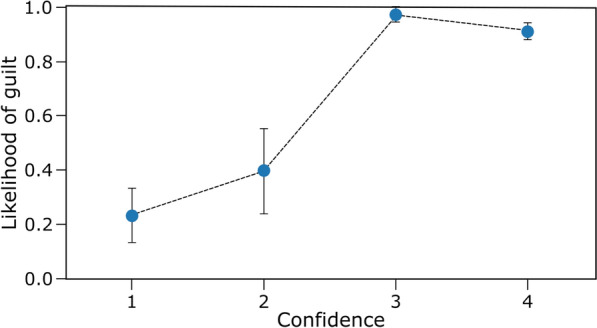
Confidence–accuracy characteristic curve for study 2, with standard error bars

Figure 8 shows the CAC curve, including rejections (rating 1), which are typically not plotted but illustrate that high-confidence rejections indicate the suspect’s innocence. Two patterns are notable. First, confidence was positively related to evidentiary strength: Identifications made with low confidence (rating of 2) were less likely to reflect high evidentiary strength than those made with moderate (3) or high (4) confidence. Second, there was little difference in the likelihood of guilt between moderate (3) and high (4) confidence, indicating that accuracy did not increase appreciably from moderate to the highest confidence level.

The ANRI for the full range of confidence ratings (1–4) was 0.41, indicating good resolution. When restricted to identification decisions (confidence levels 2–4), the ANRI was 0.22, consistent with previously reported values for fair simultaneous lineups (e.g., Carlson et al., 2016; Palua et al., 2024). The smaller value reflects the narrower range of guilt likelihood when rejections are excluded. These findings reinforce the CAC results by demonstrating that higher eyewitness confidence corresponded with stronger corroborating evidence of guilt in real-world showups.

We next examined the relationship between confidence and evidentiary strength ratings as a continuous measure. Confidence (1–4) and evidentiary strength (1–5) were strongly correlated, *r*(152) = 0.56, *R*^2^ = 0.316, a large effect size (Cohen, 1988), and above the threshold suggested by Wixted and G. Wells (2017) for a “very strong confidence-accuracy relationship” (p. 22).

### Discussion

In Study 2, we examined the confidence–accuracy relationship in real-world showups using archival data from two US law enforcement agencies. Our findings show that, consistent with the laboratory results from Study 1, eyewitness confidence is positively associated with the likelihood that a suspect is guilty (i.e., supported by strong corroborating evidence). High-confidence identifications were generally paired with stronger non-eyewitness evidence, whereas low-confidence identifications were associated with weaker evidence. These results suggest that, even in applied field settings with complexities and uncertainties of actual investigations, confidence can provide meaningful information about the predictive value of a showup identification.

An additional finding of note is that accuracy did not meaningfully increase from moderate (confidence = 3) to high confidence (confidence = 4). This plateau differs from many laboratory studies, where the highest levels of confidence are often associated with the greatest accuracy. One possible interpretation is that, in real-world showups, moderate to high confidence may already reflect a strong memory signal, leaving limited room for further gains at the highest confidence level. Alternatively, this pattern may reflect variability in how eyewitnesses use verbal confidence scales in applied settings. Importantly, this finding suggests that moderately high-confidence identifications (“probably the person”) may carry probative value similar to that of very high-confidence identifications (“definitely the person”) in field contexts, though this conclusion should be interpreted cautiously and warrants further investigation.

### Limitations

The primary limitation of this field study was the incomplete documentation of showup cases. Some missing information may have been captured by body-worn camera footage, which was not accessible to researchers. Future studies should prioritize jurisdictions where access to such recordings is allowable to provide more comprehensive data.

Another limitation was the lower-than-expected number of usable cases, which reduced statistical power despite testing only one of the two original hypotheses. Smaller sample sizes can inflate effect size estimates. However, the strong relationships observed between confidence and evidentiary strength suggest that the main findings are robust despite the reduced sample size.

Finally, because the field data were both archival and not consistently reported, it was not possible to obtain measures of response time. If feasible, future studies should prospectively collect showup data with standardized procedures for recording both confidence and response time. Doing so would allow for a stronger test of the response time–accuracy relationship in the field and provide a more complete picture of eyewitness performance in real-world showups.

### Conclusion

In Study 2, we examined the confidence–accuracy relationship in field showup identifications using SES ratings as a proxy for ground truth. Across two jurisdictions and three complementary analysis approaches (CAC, correlation, and calibration (ANRI) analyses) the results converged to show that eyewitness confidence is a meaningful indicator of accuracy in real-world showups. Specifically, CAC analysis revealed the expected positive relationship between confidence and the likelihood of guilt, with higher-confidence ratings being associated with stronger corroborating evidence. Calibration analysis further demonstrated that confidence provided moderate discrimination between likely guilt and likely innocent identifications.

Although there was no incremental increase in accuracy between moderately high and very high confidence, both levels were predictive of guilt, suggesting that high-confidence identifications in showups can be probative even without absolute certainty. Importantly, low-confidence identifications were not strongly associated with guilt, reinforcing the value of capturing confidence at the time of the initial showup. Together, these findings provide field-based support for the utility of confidence in showups, while also underscoring the need for careful administration and corroborating evidence to ensure reliability.

## General discussion

The findings from Study 1 and Study 2 converge to show that confidence is an indicator of accuracy in live showups. Study 1 (the laboratory study) demonstrated that faster and higher-confidence identifications were more accurate. This study also showed that confidence in a showup rejection was indicative of innocence, where high confidence in a rejection was more accurate than lower rejection confidence levels. Study 2 (the field study) replicated the confidence–accuracy relationship in real-world cases using the SES as a proxy measure of ground truth. Due to practical constraints in real-world procedures, response time could not be captured in the field, and thus, the response time–accuracy relationship could not be assessed. Nevertheless, the consistency of the confidence–accuracy relationship across both laboratory (controlled) and field (applied) settings highlights the value of capturing confidence statements in live showups.

While the DFD theory explains confidence in lineups and image-based showups, it does not fully account for accuracy in live showups. Our findings suggest that factors such as contextual cues, encoding specificity (Tulving & Thompson, 1973), and ecological validity may contribute to the reliability of eyewitness memory in live showups, highlighting the need for theories that address real-world, single-suspect procedures.

Another issue worth addressing is why our results differ from those reported by Eisen et al. (2022), who found weaker confidence–accuracy relationships in live showups than we report here, and more similar to those in image-based showups. One likely explanation is methodological. The procedures used in Eisen et al. were notably more suggestive, often involving highly leading showups that failed to minimize biasing influences. Suggestiveness in administering a showup can undermine the value of confidence because the procedure itself contaminates the memory signal on which confidence is based. In contrast, the present studies used showups that were administered under more controlled and less suggestive conditions. The stronger confidence–accuracy relationship observed here, therefore, underscores the importance of adhering to proper administration protocols. These results demonstrate that the utility of confidence in showups is not fixed but contingent upon the procedure: When showups are conducted in ways that reduce suggestibility and preserve memory integrity, confidence serves as a reliable indicator of accuracy.

A persistent gap between laboratory research and law enforcement practice has limited the application of empirical findings to real-world showups. Much of the research has focused on image-based procedures, which differ substantially from the live, dynamic context in which showups occur in the field. Our studies may help narrow this gap by testing the confidence–accuracy relationship under conditions that more closely approximate, or directly reflect, actual police practice. By aligning methods with real-world procedures, the present findings offer stronger guidance for policy development and training than prior laboratory-only studies.

Practically, the results support continued use of live showups when procedures minimize suggestibility and bias. High-confidence identifications aligned with stronger corroborating evidence, indicating that confidence can guide investigative decisions. Low-confidence identifications are less probative, helping agencies avoid overreliance on lower-confidence identifications. Showups should complement other evidence, serving as an initial or supportive investigative tool.

Overall, the convergence of laboratory and field results provides an empirical basis for policy and practice improvements. Agencies can train officers to consistently collect confidence statements (and response times), develop evidence-based guidelines for live showups, and incorporate confidence in evaluating identifications. Replication across additional studies would further confirm the utility of live showups in both controlled and real-world settings.

## Data Availability

Study 1 data and analysis code and Study 2 data are available here: https://osf.io/9rhkv/overview?view_only=215e9c25f6674a7cb4056fb7a9e0f9c3.

## Footnotes

1 These guidelines are an official statement of the American Psychology-Law Society; however, they do “not represent the position of the American Psychological Association or any of its other Divisions or subunits.”

2 At the time of the first viewing of the suspect only.

3 The term “potential suspect” is used to underscore the important distinction between a person who may be perceived as matching a witness’s description and a person whom the police consider a suspect. Many legal and social science researchers often refer to persons temporarily detained for a showup as suspects, even though the police are unlikely to consider the individual a suspect until, at a minimum, a field-based identification is made or made with moderate to high confidence. This is reflected in many current law enforcement agency policies on showups.

4 Unfortunately, this cannot be compared to PERF’s findings as they did not provide a breakdown of showups by agency type.

5 Some have argued that this assumption is not well-supported (Amendola, Mickes et al., 2025a and 2025b; Gonzalez et al., 1993).

6 Response times have been collected in some field research (e.g., Wixted et al., 2016).

7 From a State’s Attorney’s Office.

8 The final evidentiary category of the SES is “IDENTIFICATION INFORMATION,” defined as independent corroboration of information linking the suspect to the particular incident, regardless of source. However, all cases to be evaluated were stripped of the actual identification, but evaluators were provided with all information leading up to the identification, such as the eyewitness’s description of the perpetrator, other witness statements, and the suspect's location relative to the crime scene. Therefore, the case evaluators had evidence related to the identification of the perpetrator and instructions provided to the witness, but without the identification itself (i.e., whether the witness made an identification or not, or the eyewitness’s confidence), so as not to bias the case evaluation rating.

## References

[CR1] Rogers, E. M. (1962). Diffusion of Innovations. New York: Free Press of Glencoe. https://teddykw2.wordpress.com/wp-content/uploads/2012/07/everett-m-rogers-diffusion-of-innovations.pdf

[CR2] Amendola, K. L., & Slipka, M. G. (2009). Strength of evidence scale. *Unpublished instrument. Police Foundation, Washington, DC*. https://www.policinginstitute.org/wp-content/uploads/2020/04/About-the-Strength-of-Evidence-Scale.pdf

[CR3] Amendola, K. L., Valdovinos Olson, M., Carlson, C., Gronlund, S., Mickes, L., Gao, J., & Yang, Y. (2025a). Confidence, Latency, and Accuracy in Eyewitness Identifications Made from Showups: Nationwide Survey of Policies and Practices in Eyewitness Identification, Final Report by the National Policing Institute to the National Institute of Justice, Office of Justice Programs, U.S. Department of Justice. https://www.policinginstitute.org/publication/2025-nationwide-survey-of-eyewitness-identification-practices-among-law-enforcement-agencies/

[CR4] Amendola, K. L., Mickes, L., Valdovinos Olson, M., Carlson, C., Gronlund, S., & Chen, X. (2025b). Confidence, Latency, and Accuracy in Eyewitness Identifications Made from Showups: *Evidence from a Laboratory Experiment and Field Study.* Final Report by the National Policing Institute to the National Institute of Justice, Office of Justice Programs, U.S. Department of Justice. https://www.policinginstitute.org/publication/eyewitness-confidence-in-showups-results-from-the-lab-and-field-studies/

[CR5] Amendola, K. L., & Wixted, J. T. (2015). Comparing the diagnostic accuracy of suspect identifications made by actual eyewitnesses from simultaneous and sequential lineups in a randomized field trial. *Journal of Experimental Criminology,**11*, 263–284. 10.1007/s11292-014-9219-2

[CR55] Behrman, B. W., & Davey, S. L. (2001). Eyewitness identification in actual criminal cases: An archival analysis. *Law and human behavior*, *25*(5), 475–491. 10.1023/A:1012840831846 10.1023/a:1012840831846 11688369

[CR56] Bertelsman, S. P. (2022). Defending due process: The Case for Abolishing the Show-Up Line-Up. *Wash. UJL & Pol'y*, *68*, 245.

[CR6] Blomberg. T. G. (2021). Translational Criminology and Politics Presentation. Justice Research and Statistics Association’s (JRSA) Virtual Training. Florida State University. https://criminology.fsu.edu/sites/g/files/upcbnu3076/files/JRSA%20Blomberg%202021_11-30-21.pdf

[CR7] Brewer, N., Caon, A., & Todd, C. (2006). Eyewitness identification accuracy and response latency. *Law and Human Behavior,**30*, 31–50. 10.1007/s10979-006-9002-7 16729207 10.1007/s10979-006-9002-7

[CR57] Brooks, N. (1983). *Police guidelines: Pretrial eyewitness identification procedures*. Ottawa: Law Reform Commission of Canada. https://www.ojp.gov/ncjrs/virtual-library/abstracts/police-guidelines-pretrial-eyewitness-identificationprocedures

[CR8] Carlson, C. A., Hemby, J. A., Wooten, A. R., et al. (2021). Testing encoding specificity and the diagnostic feature-detection theory of eyewitness identification, with implications for showups, lineups, and partially disguised perpetrators. *Cognitive Research: Principles & Implications,**6*, 14. 10.1186/s41235-021-00276-3 33660118 10.1186/s41235-021-00276-3 PMC7930176

[CR9] Carlson, C. A., Lockamyeir, R. F., Carlson, M. A., Goodsell, C. A., Jones, A. R., Wooten, A. R., & Brand, Z. T. (2025). Comparing the strength of the confidence-accuracy versus response time-accuracy relationship for eyewitness identification. *Scientific Reports,**15*, 11064. 10.1038/s41598-025-96224-y 40169860 10.1038/s41598-025-96224-y PMC11962128

[CR10] Carlson, C. A., Young, D. F., Weatherford, D. R., Carlson, M. A., Bednarz, J. E., & Jones, A. R. (2016). The influence of perpetrator exposure time and weapon presence/timing on eyewitness confidence and accuracy. *Applied Cognitive Psychology,**30*(6), 898–910. 10.1002/acp.3275

[CR58] Cohen, J. (1988). The effect size. *Statistical power analysis for the behavioral sciences*, 77–83.

[CR11] Colloff, M. F., & Wixted, J. T. (2020). Why are lineups better than showups? A test of the filler siphoning and enhanced discriminability accounts. *Journal of Experimental Psychology,**26*, 124–143. 10.1037/xap0000218 30883151 10.1037/xap0000218

[CR12] Dunning, D., & Perretta, S. (2002). Automaticity and eyewitness accuracy: A 10-to 12-second rule for distinguishing accurate from inaccurate positive identifications. *Journal of Applied Psychology,**87*(5), 951. 10.1037/0021-9010.87.5.951 12395819 10.1037/0021-9010.87.5.951

[CR13] Eisen, M. L., Smith, A. M., Olaguez, A. P., & Skerritt-Perta, A. S. (2017). An examination of showups conducted by law enforcement using a field-simulation paradigm. *Psychology, Public Policy, and Law,**23*, 1–22. 10.1037/law0000115

[CR14] Eisen, M. L., Ying, R. C., Chui, C., & Swaby, M. (2022). Comparing witness performance in the field versus the lab: How real-world conditions affect eyewitness decision-making. *Law and Human Behavior,**46*, 175–188. 10.1037/lhb0000485 35604705 10.1037/lhb0000485

[CR15] Fitzgerald, R. J., Price, H. L., & Valentine, T. (2018). Eyewitness identification: Live, photo, and video lineups. *Psychology, Public Policy, and Law,**24*(3), 307–325. 10.1037/law0000164 10.1037/law0000164 PMC6078069 30100702

[CR16] Gonzalez, R., Ellsworth, P. C., & Pembroke, M. (1993). Response biases in lineups and showups. *Journal of Personality and Social Psychology,**64*(4), 525.

[CR17] Gould, J. B., Carrano, J., Leo, R., & Young, J. (2012). *Predicting Erroneous Convictions: A Social Science Approach to Miscarriages of Justice. (Report for Award No. 2009-IJ-CX-4110).* Washington, DC: National Institute of Justice, Office of Justice Programs, U.S. Department of Justice. https://www.ojp.gov/pdffiles1/nij/grants/241389.pdf

[CR18] Green, L. W., Ottoson, J. M., Garcia, C., & Hiatt, R. A. (2009). Diffusion theory and knowledge dissemination, utilization, and integration in public health. *Annual Review of Public Health,**30*(1), 151–174. 10.1146/annurev.publhealth.031308.100049 19705558 10.1146/annurev.publhealth.031308.100049

[CR19] Gronlund, S. D., Carlson, C. A., Neuschatz, J. S., Goodsell, C. A., Wetmore, S. A., Wooten, A., & Graham, M. (2012). Showups versus lineups: An evaluation using ROC analysis. *Journal of Applied Research in Memory and Cognition,**1*, 221–228. 10.1016/j.jarmac.2012.09.003

[CR20] Holdstock, J. S., Dalton, P., May, K., Boogert, S., & Mickes, L. (2022). Lineup identification in young and older witnesses: Does describing the criminal help or hinder?”. *Cognitive Research: Principles and Implications*. 10.1186/s41235-022-00399-1 35713818 10.1186/s41235-022-00399-1 PMC9206054

[CR21] Juslin, P., Olsson, N., & Winman, A. (1996). Calibration and diagnosticity of confidence in eyewitness identification: Comments on what can be inferred from the low confidence-accuracy correlation. *Journal of Experimental Psychology: Learning, Memory, and Cognition,**22*, 1304–1316. 10.1037/0278-7393.22.5.1304

[CR22] Katzman, J. A., & Kovera, M. B. (2022). Evidence strength (insufficiently) affects police officers’ decisions to place a suspect in a lineup. *Law and Human Behavior,**46*(1), 30–44. 10.1037/lhb0000476 34968099 10.1037/lhb0000476

[CR23] Key, K. N., Cash, D. K., Neuschatz, J. S., Price, J., Wetmore, S. A., & Gronlund, S. D. (2015). Age differences (or lack thereof) in discriminability for lineups and showups. *Psychology, Crime & Law,*. 10.1080/1068316X.2015.1054387

[CR24] Mickes, L. (2015). Receiver operating characteristic analysis and confidence-accuracy characteristic analysis in investigations of system variables and estimator variables that affect eyewitness memory. *Journal of Applied Research in Memory and Cognition,**4*, 93–102. 10.1016/j.jarmac.2015.01.003

[CR25] Mickes, L., Hwe, V., Wais, P. E., & Wixted, J. T. (2011). Strong memories are hard to scale. *Journal of Experimental Psychology: General,**140*, 239–257. 10.1037/a0023007 21417544 10.1037/a0023007

[CR26] Mickes, L., Seale-Carlisle, T. M., Chen, X., & Boogert, S. (2023). pyWitness 1.0: A Python eyewitness identification analysis toolkit. *Behavior Research Methods*. 10.3758/s13428-023-02108-2 37540469 10.3758/s13428-023-02108-2 PMC10991016

[CR27] Morris, Z. S., Wooding, S., & Grant, J. (2011). The answer is 17 years, what is the question: Understanding time lags in translational research. *Journal of the Royal Society of Medicine,**104*(12), 510–520. 10.1258/jrsm.2011.110180 22179294 10.1258/jrsm.2011.110180 PMC3241518

[CR28] National Research Council. (2014). *Identifying the Culprit: Assessing Eyewitness Identification*. The National Academies Press.

[CR29] Norman, D. A. (2010). The research-practice gap: The need for translational developers. *Interactions,**17*(4), 9–12. 10.1145/1806491.1806494

[CR30] Palua, A., Raidveea, A., Murnikovb, V., & Kask, K. (2024). The effect of surgical masks on identification decisions from masked and unmasked lineups. *Psychiatry, Psychology and Law,**31*(5), 932–962. 10.1080/13218719.2023.2242435 10.1080/13218719.2023.2242435 PMC11418056 39318879

[CR31] Police Executive Research Forum. (PERF, 2013). A National Survey of Eyewitness Identification Procedures in Law Enforcement Agencies. Retrieved from http://policeforum.org/library/eyewitness-identification/NIJEyewitnessReport.pdf

[CR32] Sauerland, M., Sagana, A., Sporer, S. L., & Wixted, J. T. (2018). Decision time and confidence predict choosers’ identification performance in photographic showups. *PLoS ONE,**13*(1), Article e0190416. 10.1371/journal.pone.0190416 29346394 10.1371/journal.pone.0190416 PMC5773080

[CR33] Seale-Carlisle, T. M., Colloff, M. F., Flowe, H. D., Wells, W., Wixted, J. T., & Mickes, L. (2019). Confidence and response time as indicators of eyewitness identification accuracy in the lab and in the real world. *Journal of Research in Memory and Cognition,**8*, 420–428. 10.1016/j.jarmac.2019.09.003

[CR34] Seale-Carlisle, T. M., Quigley-McBride, A., Teitcher, J. E. F., Crozier, W. E., Dodson, C. S., & Garrett, B. L. (2024). New insights on expert opinion about eyewitness memory research. *Perspectives on Psychological Science*. 10.1177/17456916241234837 38635239 10.1177/17456916241234837 PMC12408934

[CR59] Seale-Carlisle, T. M., Wilson, B. M., Yilmaz, A. S., Wixted, J. T., Semmler, C., & Mickes, L. (2026). The effects of instructions on performance in lineups and showups. *Journal of Experimental Psychology: Applied*. 10.1037/xap0000567 41910561 10.1037/xap0000567

[CR35] Stemler, S. E. (2004). A comparison of consensus, consistency, and measurement approaches to estimating interrater reliability. *Practical Assessment, Research, and Evaluation*. 10.7275/96jp-xz07

[CR36] Stoddard, G. D. (1935). The Lag between Practice and Research. *The Phi Delta Kappan,**17*(6), 180–182.

[CR37] Suggs, D. L. (1979). The use of psychological research by the judiciary: Do the courts adequately assess the validity of the research? *Law and Human Behavior,**3*(1–2), 135. 10.1007/BF01039153

[CR38] Tekin, E., DeSoto, K. A., Wixted, J. H., & Roediger, H. L. (2021). Applying confidence accuracy characteristic plots to old/new recognition memory experiments. *Memory,**29*, 427–443. 10.1080/09658211.2021.1901937 33826482 10.1080/09658211.2021.1901937

[CR39] Tulving, E., & Thomson, D. (1973). Encoding specificity and retrieval processes in episodic memory (1973). *Psychological Review,**80*(5), 352–373. 10.1037/h0020071

[CR40] Valentine, T., Davis, J. P., Memon, A., & Roberts, A. (2012). Live showups and their influence on a subsequent video lineup. *Applied Cognitive Psychology,**26*, 1–23. 10.1002/acp.1796

[CR41] Wells, G. L., Kovera, M. B., Douglass, A. B., Brewer, N., Meissner, C. A., & Wixted, J. T. (2020). Policy and procedure recommendations for the collection and preservation of eyewitness identification evidence. *Law and Human Behavior,**44*(1), 3–36. 10.1037/lhb0000359 32027160 10.1037/lhb0000359

[CR42] Wells, W., Campbell, B., Li, Y., & Swindle, S. (2016). The characteristics and results of eyewitness identification procedures conducted during robbery investigations in Houston. *TX. Policing: An International Journal,**39*(4), 601–619. 10.1108/PIJPSM-10-2015-0124

[CR43] Wetmore, S. A., Neuschatz, J. S., Gronlund, S. D., Wooten, A., Goodsell, C. A., & Carlson, C. A. (2015). Effect of retention interval on showup and lineup performance. *Journal of Applied Research in Memory and Cognition,**4*, 8–14. 10.1016/j.jarmac.2014.07.003

[CR44] Wilson, B. M., Seale-Carlisle, T. M., & Mickes, L. (2018). The effects of verbal descriptions on performance in lineups and showups. *Journal of Experimental Psychology: General,**147*(1), 113–124. 10.1037/xge0000354 28872330 10.1037/xge0000354

[CR45] Wixted, J. T., & Mickes, L. (2014). A signal-detection-based diagnostic-feature detection model of eyewitness identification. *Psychological Review,**121*, 262–276. 10.1037/a0035940 24730600 10.1037/a0035940

[CR46] Wixted, J. T., Mickes, L., Clark, S. E., Gronlund, S. D., & Roediger, H. (2015). Initial eyewitness confidence reliably predicts eyewitness identification accuracy. *American Psychologist,**70*, 515–526. 10.1037/a0039510 26348334 10.1037/a0039510

[CR47] Wixted, J. T., Mickes, L., Dunn, J. C., Clark, S. E., & Wells, W. (2016). Estimating the reliability of eyewitness identifications from police lineups, Proceedings of the National Academy of Sciences. *USA,**113*(2), 304–309. 10.1073/pnas.1516814112 PMC4720310 26699467

[CR49] Wixted, J. T., & Wells, G. L. (2017). The relationship between eyewitness confidence and identification accuracy: A new synthesis. *Psychological Science in the Public Interest,**18*, 10–65. 28395650 10.1177/1529100616686966

[CR50] Yaniv, I., Yates, J. F., & Smith, J. K. (1991). Measures of discrimination skill in probabilistic judgment. *Psychological Bulletin,**110*(3), 611.

[CR51] Yarmey, A. D., Yarmey, A. L., & Yarmey, M. J. (1994). Face and voice identifications in showups and lineups. *Applied Cognitive Psychology,**8*, 453–464.

[CR52] Yarmey, A. D., Yarmey, M. J., & Yarmey, A. L. (1996). Accuracy of eyewitness identifications in showups and lineups. *Law and Human Behavior,**20*(4), 459–477. 10.1007/BF01498981

[CR53] Yates, S. Q. (2017, January 6). *Eyewitness identification: Procedures for conducting photo arrays* (Memorandum). U.S. Department of Justice. https://www.justice.gov/archives/opa/press-release/file/923201/dl?inline

[CR54] Yilmaz, A. S., Wang, X., & Wixted, J. T. (2024). Response bias modulates the confidence-accuracy relationship for both positive identifications and lineup rejections in a simultaneous lineup task. *Applied Cognitive Psychology,**38*(2), Article e4196. 10.1002/acp.4196

